# Arthritogenic T cell epitope in glucose-6-phosphate isomerase-induced arthritis

**DOI:** 10.1186/ar2545

**Published:** 2008-11-07

**Authors:** Keiichi Iwanami, Isao Matsumoto, Yoko Tanaka, Asuka Inoue, Daisuke Goto, Satoshi Ito, Akito Tsutsumi, Takayuki Sumida

**Affiliations:** 1 Department of Clinical Immunology, Doctoral Program in Clinical Sciences, Graduate School of Comprehensive Human Science, University of Tsukuba, 1-1-1 Tennoudai, Tsukuba 305-8575, Japan; 2 PRESTO, Japan Science and Technology Agency, 4-1-8 Honcho Kawaguchi, Saitama 332-0012, Japan

## Abstract

**Introduction:**

Arthritis induced by immunisation with glucose-6-phosphate isomerase (GPI) in DBA/1 mice was proven to be T helper (Th) 17 dependent. We undertook this study to identify GPI-specific T cell epitopes in DBA/1 mice (H-2q) and investigate the mechanisms of arthritis generation.

**Methods:**

For epitope mapping, the binding motif of the major histocompatibility complex (MHC) class II (I-Aq) from DBA/1 mice was identified from the amino acid sequence of T cell epitopes and candidate peptides of T cell epitopes in GPI-induced arthritis were synthesised. Human GPI-primed CD4+ T cells and antigen-presenting cells (APCs) were co-cultured with each synthetic peptide and the cytokine production was measured by ELISA to identify the major epitopes. Synthetic peptides were immunised in DBA/1 mice to investigate whether arthritis could be induced by peptides. After immunisation with the major epitope, anti-interleukin (IL) 17 monoclonal antibody (mAb) was injected to monitor arthritis score. To investigate the mechanisms of arthritis induced by a major epitope, cross-reactivity to mouse GPI peptide was analysed by flow cytometry and anti-GPI antibodies were measured by ELISA. Deposition of anti-GPI antibodies on the cartilage surface was detected by immunohistology.

**Results:**

We selected 32 types of peptides as core sequences from the human GPI 558 amino acid sequence, which binds the binding motif, and synthesised 25 kinds of 20-mer peptides for screening, each containing the core sequence at its centre. By epitope mapping, human GPI325–339 was found to induce interferon (IFN) γ and IL-17 production most prominently. Immunisation with human GPI325–339 could induce polyarthritis similar to arthritis induced by human GPI protein, and administration of anti-IL-17 mAb significantly ameliorated arthritis (p < 0.01). Th17 cells primed with human GPI325–339 cross-reacted with mouse GPI325–339, and led B cells to produce anti-mouse GPI antibodies, which were deposited on cartilage surface.

**Conclusions:**

Human GPI325–339 was identified as a major epitope in GPI-induced arthritis, and proved to have the potential to induce polyarthritis. Understanding the pathological mechanism of arthritis induced by an immune reaction to a single short peptide could help elucidate the pathogenic mechanisms of autoimmune arthritis.

## Introduction

Rheumatoid arthritis (RA) is characterised by symmetrical polyarthritis and joint destruction. Although the aetiology is considered to be autoimmune reactivity to some antigens, the exact mechanisms are not fully understood. So far, several models of arthritis have been described and analysed to understand the aetiological mechanisms of RA. Glucose-6-phosphate isomerase (GPI)-induced arthritis, a murine model of RA, is induced by immunisation with recombinant human (rh) GPI of DBA/1 mice [1]. We have previously demonstrated that the T helper (Th) 17 subset of CD4^+ ^T cells play a central role in the pathogenesis of GPI-induced arthritis; GPI-specific CD4^+ ^T cells were skewed to Th17 at the time of onset, and blockade of interleukin (IL) 17 resulted in a significant amelioration of arthritis [2]. Furthermore, the data that the administration of cytotoxic T-lymphocyte antigen 4 immunoglobulin (CTLA-4 Ig) in the effector phase ameliorated the progress of arthritis implies the importance of Th17 cells even in the effector phase [3].

In this study, we further explored the epitopes of GPI-specific CD4^+ ^T cells and identified human GPI (hGPI)_325–339 _as a major epitope. Interestingly, the amino acid sequence of hGPI_325–339 _(IWYINCFGCETHAML) was the same as that of bovine (type II collagen) CII_256–270_(GEPGIAGFKGEQGPK), the dominant epitope of collagen-induced arthritis (CIA), at the major histocompatibility complex (MHC) binding sites [4]. Of note is that arthritis similar to GPI-induced arthritis was generated by immunisation with a short 15-mer single peptide in genetically unaltered mice. By analysis of peptide-induced arthritis, we found that hGPI_325–339_-primed Th17 cells reacted with mouse GPI (mGPI)_325–339 _peptide and subsequently lead to the production of anti-mouse GPI antibodies, which deposited over the cartilage surface of inflaming joints. Our findings should be helpful in unravelling the mechanism of autoimmune arthritis.

## Materials and methods

### Mice

DBA/1 mice were purchased from Charles River Laboratories, Japan. All mice were kept under specific pathogen-free conditions and all experiments were conducted in accordance with the University of Tsukuba ethical guidelines.

### GPI and synthetic peptides

Recombinant mouse GPI and rhGPI were prepared as described previously [5,6]. Briefly, human GPI or mouse GPI cDNA was inserted into the plasmid pGEX-4T3 (Pharmacia, Uppsala, Sweden) for expression of glutathione S-transferase-tagged proteins. *Escherichia coli *harboring the pGEX-hGPI plasmid was allowed to proliferate at 37°C, before 0.1 mM isopropyl-β-D-thiogalactopyranoside was added to the medium, followed by further culture overnight at 30°C. The bacteria were lysed with a sonicator and the supernatant was purified with a glutathione-sepharose column (Pharmacia, Uppsala, Sweden). The purity was estimated by SDS-PAGE.

Crude peptides were synthesised for epitope screening by Mimotopes (Melbourne, Victoria, Australia), and peptides with 90% purity were synthesised for a major epitope decision and induction of arthritis by Invitrogen (Carlsbad, CA). Candidate peptides, which were thought to bind the binding motif, were selected with web soft MHCPred (The Jenner Institute, Oxford, UK) [7].

### Induction of arthritis

DBA/1 mice were immunised with 300 μg rhGPI for GPI-induced arthritis, or 10 μg or 25 μg synthetic peptide for peptide-induced arthritis in complete Freund's adjuvant (Difco Laboratories, Detroit, MI). The rhGPI and synthetic peptide were emulsified with complete Freund's adjuvant at a 1:1 ratio (v/v). For induction of arthritis, 150 μl of the emulsion was injected intradermally at the base of the tail of the mouse. On days 0 and 2 after immunisation, 200 ng of pertussis toxin was injected intraperitoneally to develop peptide-induced arthritis. The arthritis score was evaluated visually using a score of 0 to 3 for each paw. A score of 0 represented no evidence of inflammation, 1 represented subtle inflammation or localised oedema, 2 represented easily identified swelling but localised to either the dorsal or ventral surface of the paws, and 3 represented swelling in all areas of the paws.

### Treatments of arthritis with anti-IL-17 monoclonal antibodies

To neutralise IL-17, mice were injected intraperitoneally with 100 μg of neutralising antibody or isotype control on day 7 or day 6, 8, and 10. Anti-IL-17 mAb MAB421 (IgG2a) was purchased from R&D Systems (Minneapolis, MN, USA). IgG2a isotype control was purchased from eBioscience (San Diego, CA, USA).

### Analysis of cytokine production

Mice were sacrificed on the indicated day. Spleens were harvested and haemolysed with a solution of 0.83% NH_4_Cl, 0.12% NaHCO_3 _and 0.004% EDTA_2_Na in PBS. Single-cell suspensions were prepared in RPMI1640 medium (Sigma-Aldrich, St. Louis, MO) containing 10% FCS, 100 U/ml of penicillin, 100 μg/ml of streptomycin and 50 μM 2-mercaptoethanol. CD4^+ ^T cells were isolated by MACS positive selection (Miltenyi Biotec, Bergisch Gladbach, Germany). The purity of the collected cells (>97%) was confirmed by flow cytometry. Splenic feeder cells treated with 50 μg/ml of mitomycin C were used as antigen presenting cells (APCs). The purified CD4^+ ^T cells and APCs were co-cultured with 10 μM of the synthetic peptide at a ratio of 5:1 at 37°C under 5% CO_2 _for 24 hours. The supernatants were assayed for interferon (IFN)-γ and IL-17 by Quantikine ELISA kit (R&D Systems, Minneapolis, MN).

### Intracellular cytokine staining and flow cytometric analysis

Mice were sacrificed on day 5. The draining lymph nodes were harvested and single cell suspensions were prepared as described above. Cells (1×10^6^/ml) were stimulated with 10 μM of the synthetic peptides in 96-well round bottom plates (Nunc, Roskilde, Denmark) for 24 hours and GoldiStop (BD PharMingen, San Diego, CA) was added for the last four hours of each culture. Cells were first stained extracellularly, fixed and permeabilised with Cytofix/Cytoperm solution (BD PharMingen, San Diego, CA) and then stained intracellularly. Samples were acquired on FACSCalibur (BD PharMingen, San Diego, CA) and data were analysed with FlowJo (Tree Star, Ashland, OR).

### Analysis of anti-GPI antibody

Sera were taken from immunised mice on day 14 and diluted 1:500 in blocking solution (25% Block Ace (Dainippon Sumitomo Pharma, Osaka, Japan) in PBS) for antibody analysis. We also prepared 96-well plates (Sumitomo Bakelite, Tokyo, Japan) coated with 5 μg/ml rhGPI or recombinant mouse GPI for 12 hours at 4°C. After washing twice with a washing buffer (0.05% Tween20 in PBS), the blocking solution was used for blocking nonspecific binding for two hours at room temperature. After three washes, 150 μl of the diluted serum was added and incubated for two hoursat room temperature. After three washes, alkaline phosphatase-conjugated anti-mouse IgG was added at a final dilution of 1:5000, for one hour at room temperature. After three washes, colour was developed with substrate solution (1 alkaline phosphatase tablet (Sigma-Aldrich, St. Louis, MO, USA) per 5 ml alkaline phosphatase reaction solution (containing 9.6% diethanolamine and 0.25 mM MgCl_2_, pH 9.8)). Plates were incubated for 20 minutes at room temperature and optical density was measured by a microplate reader at 405 nm.

### Immunohistology

For immunohistology, cryostat sections from ankle joints were prepared with the tape capture technique as described previously [8]. Briefly, ankle joints were taken from immunised mice on day 14 and placed in Tissue-Tek (Sakura Finetek, Torrance, CA) filled with 4% carboxymethyl cellulose compound (Finetec, Tokyo, Japan). Frozen ankles joints in the carboxymethyl cellulose compound were attached to the adhesive Cryofilm (Finetec, Tokyo, Japan) and were cut in the microtome. The sections on the adhesive film were fixed with cold acetone. After blocking with 2% bovine serum albumin and 0.05% Tween in PBS, the sections were stained with Alexa 546-conjugated anti-mouse IgG (Invitrogen, Carlsbad, CA) (200 ng/slide), and nuclei were counterstained with 4',6-diamidino-2-phenyindole dilactate (DAPI) (Sigma-Aldrich, St. Louis, MO, USA) (50 ng/slide). Fluorescence was detected with the Leica DMRA2 microscopy (Leica, Wetzlar, Germany). The images were acquired and processed with Leica FW4000 (Leica, Wetzlar, Germany).

### Statistical analysis

All data were expressed as mean ± standard error of the mean (SEM) or standard deviation (SD). Differences between groups and variables were examined for statistical significance using the Mann-Whitney's U test and the Spearman's rank correlation coefficient, respectively. A p < 0.05 denoted the presence of a statistically significant difference.

## Results

### I-A^q ^binding motif and epitope candidates

To analyse T cell epitopes, we first investigated the binding motif of I-A^q ^from T cell epitopes reported in the literature because DBA/1 mice express only I-A^q ^as MHC class II. Based on the work by Bayrak and colleagues [9], the anchor motif of I-A^q ^would exist at P1, P4 and P7, therefore we predicted the binding motifs from amino acid sequences of I-A^q ^restricted epitopes on murine RNase_90–105 _[10], myelin basic protein_89–101 _[11,12], chicken type II collagen (CII)_181–209 _[13], rat CII_256–270 _[14,15], bovine CII_256–270 _[4] and mouse type II collagen [9] (Table 1). Next, we selected 32 types of peptides as core sequences from the human GPI 558 amino acid sequence, which is thought to bind the binding motif (Table 2), and synthesised 25 kinds of 20-mer peptides for screening, each containing the core sequence in its centre (Table 3).

**Table 1:** I-Aq binding motifs

P1	P2	P3	P4	P5	P6	P7	P8	P9
**A**			**A**			**E**		
**F**			**P**			**D**		
**L**			**F**			**Q**		
**I**			**S**			**P**		
**P**			**V**			**N**		
**S**			**L**			**I**		
**V**			**N**					
			**R**					

The anchor motif of I-A^q ^would exist at P1, P4 and P7, therefore we predicted the binding motif from amino acid sequences of I-A^q ^restricted epitopes on murine RNase_90–105_, myelin basic protein_89–101_, chicken type II collagen_181–209_, rat type II collagen_256–270_, bovine type II collagen_256–270 _and mouse type II collagen.

**Table 2:** Core sequences of glucose-6-phosphate isomerase (GPI) amino acids binding I-A^q^

Peptide	Amino acid residues
3–11	**A**LT**R**DP**Q**FQ
29–37	**L**FD**A**NK**D**RF
41–49	**S**LT**L**NT**N**HG
56–64	**S**KN**L**VT**E**DV
72–80	**A**KS**R**GV**E**AA
80–88	**A**RE**R**MF**N**GE
99–107	**L**HV**A**LR**N**RS
102–110	**A**LR**N**RS**N**TP
149–157	**I**TD**V**IN**I**GI
167–175	**V**TE**A**LK**P**YS
173–181	**P**YS**S**GG**P**RV
181–189	**V**WY**V**SN**I**DG
196–204	**L**AQ**L**NP**E**SS
201–209	**P**ES**S**LF**I**IA
210–218	**S**KT**F**TT**Q**ET
229–237	**F**LQ**A**AK**D**PS
230–238	**L**QA**A**KD**P**SA
243–251	**F**VA**L**ST**N**TT
253–261	**V**KE**F**GI**D**PQ
285–293	**A**LH**V**GF**D**NF
319–327	**L**LA**L**LG**I**WY
328–336	**I**NC**F**GC**E**TH
337–345	**A**ML**P**YD**Q**YL
391–399	**F**YQ**L**IH**Q**GT
403–411	**P**CD**F**LI**P**VQ
407–415	**L**IP**V**QT**Q**HP
426–434	**L**AN**F**LA**Q**TE
452–460	**A**GK**S**PE**D**LE
489–497	**A**LV**A**MY**E**HK
537–545	**S**HD**A**ST**N**GL
540–548	**A**ST**N**GL**I**NF
545–553	**L**IN**F**IK**Q**QR

Thirty-two types of peptides were selected as core sequences from the GPI 558 amino acid sequence, which is thought to bind the binding motif. Amino acid residues that are thought to bind anchors of I-A^q ^are shown in bold letters.

**Table 3:** Synthetic peptides for screening T cell epitopes

Peptide number	Peptide	Synthetic peptide sequence
1	1–20	H-MA**A**LT**R**DP**Q**FQKLQQWYREH-OH
2	23–42	H-ELNLRR**L**FD**A**NK**D**RFNHFSL-OH
3	37–56	H-FNHF**S**LT**L**NT**N**HGHILVDYS-OH
4	51–70	H-ILVDY**S**KN**L**VT**E**DVMRMLVD-OH
5	71–90	H-L**A**KS**R**GV**E**A**A**RE**R**MF**N**GEKI-OH
6	96–115	H-RAV**L**HV**A**LR**N**RS**N**TPILVDG-OH
7	145–164	H-TGKT**I**TD**V**IN**I**GIGGSDLGP-OH
8	162–181	H-LGPLM**V**TE**A**LK**P**YSSGGPRV-OH
9	168–187	H-TEALK**P**YS**S**GG**P**RVWYVSNI-OH
10	176–195	H-SGGPR**V**WY**V**SN**I**DGTHIAKT-OH
11	191–210	H-HIAKT**L**AQ**L**NP**E**SSLFIIAS-OH
12	200–219	H-N**P**ES**S**LF**I**IA**S**KT**F**TT**Q**ETI-OH
13	225–244	H-AKEW**FL**Q**AA**K**DP**SAVAKHFV-OH
14	238–257	H-AVAKH**F**VA**L**ST**N**TTKVKEFG-OH
15	247–266	H-STNTTK**V**KE**F**GI**D**PQNMFEF-OH
16	280–299	H-IGLSI**A**LH**V**GF**D**NFEQLLSG-OH
17	313–332	H-EKNAPV**L**LA**L**LG**I**WYINCFG-OH
18	327–346	H-Y**I**NC**F**GC**E**TH**A**ML**P**YD**Q**YLH-OH
19	386–405	H-NGQHA**F**YQ**L**IH**Q**GTKMIPCD-OH
20	400–419	H-KMI**P**CD**FL**I**PV**QT**Q**HPIRKG-OH
21	420–439	H-LHHKIL**L**AN**F**LA**Q**TEALMRG-OH
22	445–464	H-ARKELQA**A**GK**S**PE**D**LERLLP-OH
23	484–503	H-PFMLG**A**LV**A**MY**E**HKIFVQGI-OH
24	533–552	H-AQVT**S**HD**A**ST**N**GL**I**NFIKQQ-OH
25	539–558	H-DASTNG**L**IN**F**IK**Q**QREARVQ-OH

Listed are 25 20-mer unpurified peptides in which each core sequence were centred around. Amino acid residues constituting the core sequence and those thought to bind anchors of I-A^q ^are underlined and shown in bold letters, respectively.

### Epitope screening

**Figure 1:**
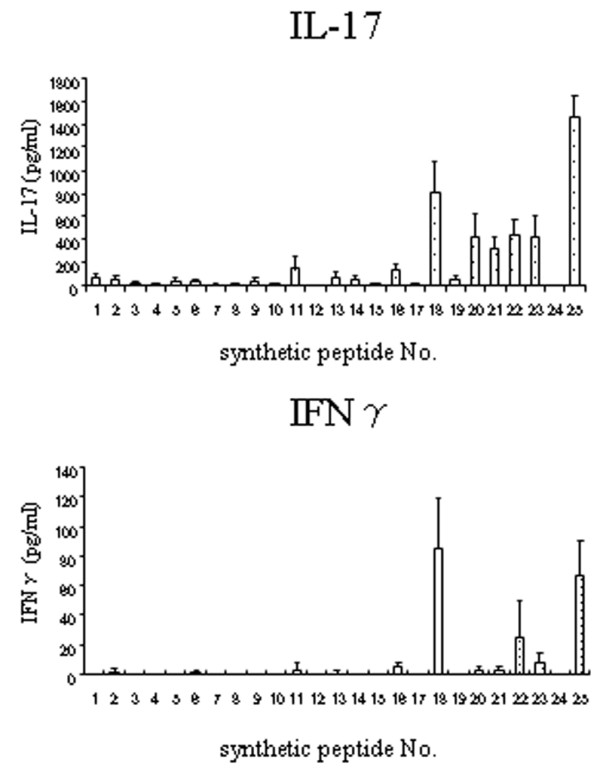
**Synthetic peptides number 18 and 25 produced marked simulation of glucose-6-phosphate isomerase (GPI) primed CD4^+ ^T cells**. Mice were sacrificed on day 7 after immunisation. CD4^+ ^T cells were purified from spleen cells of GPI-immunised DBA/1 mice. GPI-primed CD4^+ ^T cells and antigen presenting cells (APCs) were co-cultured with 10 μM of synthetic peptide for 24 hours. The supernatants were assayed for interferon (IFN) γ and interleukin (IL) 17 by ELISA. Data are averages ± standard deviation of three culture wells. Representative data of three independent experiments.

rhGPI-specific CD4^+ ^T cells differentiate into Th1 and Th17 [2], so we analysed IFN-γ and IL-17 production for epitope screening when rhGPI-primed CD4^+ ^T cells were stimulated with each synthetic peptide. The production of both IFN-γ and IL-17 was pronounced when GPI-primed CD4^+ ^T cells were stimulated with number 18 peptide (hGPI_327–346_) and number 25 peptide (hGPI_539–558_). Therefore, we considered that major epitopes exist in either of the two peptides (Figure 1). In the K/BxN mouse model of arthritis, KRN T cell receptor (TCR) transgenic T cells recognise mGPI_282–294_, the dominant epitope of K/BxN mouse, on I-A^g7 ^[16]. However, in the GPI-induced arthritis model, it was unlikely that hGPI_282–294 _was the dominant epitope because GPI-specific T cells did not react prominently to number 16 peptide (hGPI_280–299_).

**Figure 2:**
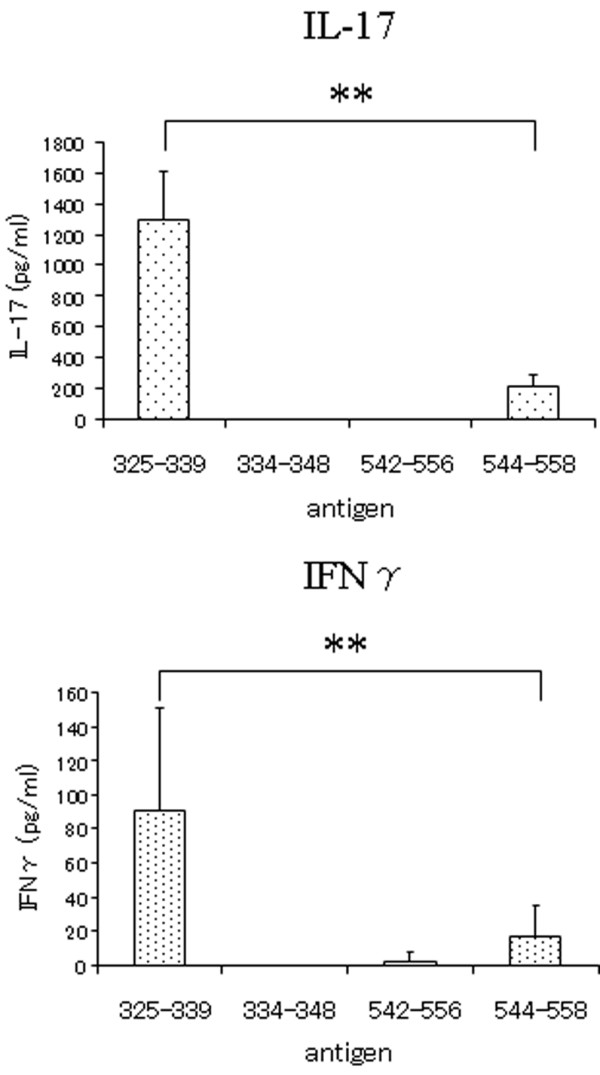
**GPI_325–339 _is a major epitope**. Mice were sacrificed on day 7 after immunisation. CD4^+ ^T cells were purified from splenocytes of glucose-6-phosphate isomerase (GPI) immunised DBA/1 mice. GPI-primed CD4^+ ^T cells and antigen presenting cells (APCs) were co-cultured with 10 μM of synthetic peptide hGPI_325–339_, hGPI_334–348_, hGPI_542–556 _or hGPI_544–558 _for 24 hours. The purity of each peptide was 90%. The supernatants were assayed for interferon (IFN) γ and interleukin (IL) 17 by ELISA. Data are averages ± standard deviation of five culture-wells. **p < 0.01 (Mann-Whitney's U test). Representative data of three independent experiments.

Because the synthetic peptides used for screening were not purified, we re-synthesised the 15-mer peptides with a purity of 90%; these peptides contained each core sequence of number 18 peptide (hGPI_327–346_) and number 25 peptide (hGPI_539–558_). Number 18 peptide (hGPI_327–346_) contains two core sequences (hGPI_328–336 _and hGPI_337–345_), so therefore we re-synthesised two peptides (hGPI_325–339 _and hGPI_334–348_). The former sequences of number 25 peptide (hGPI_539–558_) overlapped with number 24 peptide (hGPI_533–552_), which could not stimulate CD4^+ ^T cells primed with GPI. Therefore we re-synthesised two peptides (hGPI_542–556 _and hGPI_544–558_) from the latter sequences of number 25 peptide (Table 4). We analysed IFN-γ and IL-17 production for epitope screening as described above. The peptide (hGPI_325–339_) induced marked stimulation of GPI-primed CD4^+ ^T cells, and was considered a major epitope (Figure 2).

**Table 4:** Re-synthesised peptides used for determining a major epitope

Peptide number	Peptide	Synthetic peptide sequence
18	327–346	H-Y**I**NC**F**GC**E**TH**A**ML**P**YD**Q**YLH-OH
	325–339	H-IWY**I**NC**F**GC**E**THAML-OH
	334–348	H-ETH**A**ML**P**YD**Q**YLHRF-OH
		
25	539–558	H-DASTNG**L**IN**F**IK**Q**QREARVQ-OH
	542–556	H-TNG**L**IN**F**IK**Q**QREAR-OH
	544–558	H-G**L**IN**F**IK**Q**QREARVQ-OH

The 15-mer peptides were synthesised with 90% purity, containing each core sequence of number 18 peptide (GPI_327–346_) and number 25 peptide (GPI_539–558_). Amino acid residues constituting the core sequence and those thought to bind the anchors of I-A^q ^are underlined and shown in bold letters, respectively.

### Immunisation with a major epitope induces arthritis similar to GPI-induced arthritis

**Figure 3:**
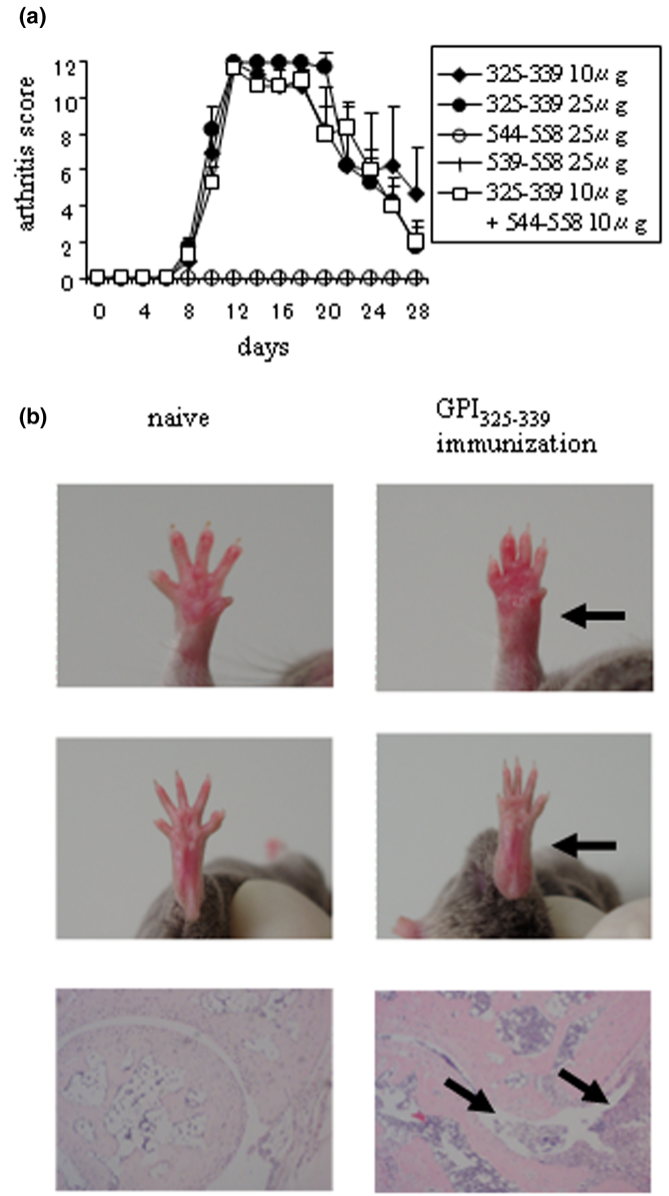
**Immunisation with hGPI_325–339 _induces severe polyarthritis**. DBA/1 mice were immunised with 25 μg of hGPI_325–339_, hGPI_539–558 _or hGPI_544–558_, or 10 μg each of hGPI_325–339 _plus hGPI_544–558_, and 200 ng of pertussis toxin was injected intraperitoneally on days 0 and 2 after immunisation. (a) The mean arthritis score (± standard error of the mean (SEM)) of five mice in one representative experiment of two independent experiments. (b) Severe swelling of the wrist (upper panels) and ankle joints (middle panels) in mice immunised with 25 μg of hGPI_325–339 _compared with naïve mice (arrowheads). Histological analysis of haematoxylin & eosin-stained sections of ankle joints taken from naïve mice and mice on day 14 after hGPI_325–339 _immunization (lower panels) showed severe synovitis with massive infiltration of cells and hyperplasia of synovial tissue (arrowheads).

To test if hGPI_325–339 _is arthritogenic, DBA/1 mice were immunised with 10 μg or 25 μg hGPI_325–339 _instead of GPI protein, and 200 ng of pertussis toxin was injected intraperitoneally on days 0 and 2 after immunisation. Arthritis resembling GPI-induced arthritis could be generated by immunisation with the peptide, including incidence, manifestations and severity. Symmetrical polyarthritis appeared on day 8, showed peak severity on day 14 and subsided gradually thereafter (Figure 3a). The use of different immunisation doses (10 and 25 μg) did not seem to affect the incidence and severity of arthritis. Immunised with 10 μg or 25 μg hGPI_325–339 _without injection of pertussis toxin could also induce arthritis. However, the arthritis was less severe than with pertussis toxin (data not shown). On the other hand, immunisation with neither hGPI_539–558 _nor hGPI_544–558_, which were considered minor epitopes in GPI-induced arthritis, could induce overt arthritis (Figure 3a). Mice immunised with hGPI_325–339 _developed severe swelling of the wrist and ankle joints. Histologically, severe synovitis was noted in the wrists in the forepaws, and at ankles and tarsal joints in the hind paws (Figure 3b and data not shown).

### Peptide-induced arthritis is mediated by Th17

**Figure 4:**
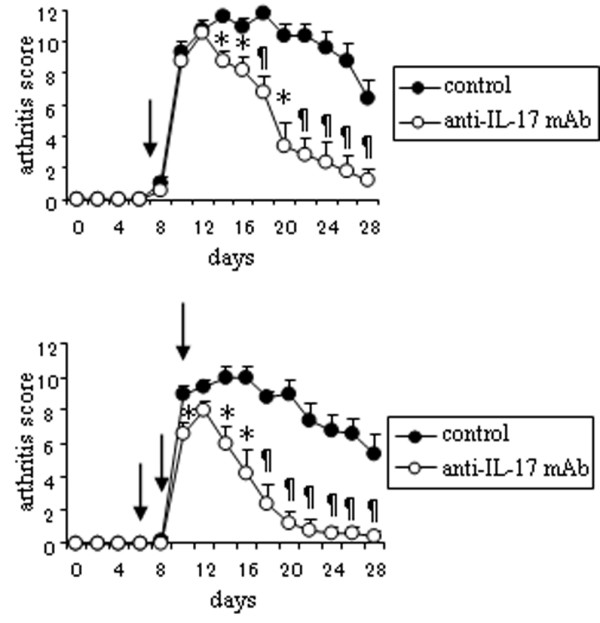
**Anti-IL-17 monoclonal antibody (mAb) suppresses the development of arthritis**. DBA/1 mice were immunised with 25 μg of hGPI_325–339_, and 200 ng of pertussis toxin was injected intraperitoneally on days 0 and 2 after immunisation. 100 μg of anti-IL-17 mAb or isotype control (control) was administered intraperitoneally on day 7 (upper panel) or day 6, 8, and 10 (lower panel) after immunisation (arrow). Mean arthritis score (± standard error of the mean (SEM)) of five mice per group. Representative data of two independent experiments. * p < 0.05, ¶p < 0.01 (Mann-Whitney's U test).

GPI-induced arthritis is Th17-mediated [2], so we explored the aetiological role of Th17 in peptide-induced arthritis. Like GPI-induced arthritis, one time administration of anti-IL-17 mAb on day 7 and three times administration on day 6, 8 and 10 significantly ameliorated the arthritis (Figure 4). From these data, the arthritis induced by hGPI_325–339 _was also considered to be Th17 mediated.

### Immunisation of human GPI_325–339 _leads Th17 cells to cross-react with mouse GPI_325–339_

We examined the pathogenesis of arthritis induced by hGPI_325–339 _by comparing it with mice immunised with hGPI_544–558_.

**Figure 5:**
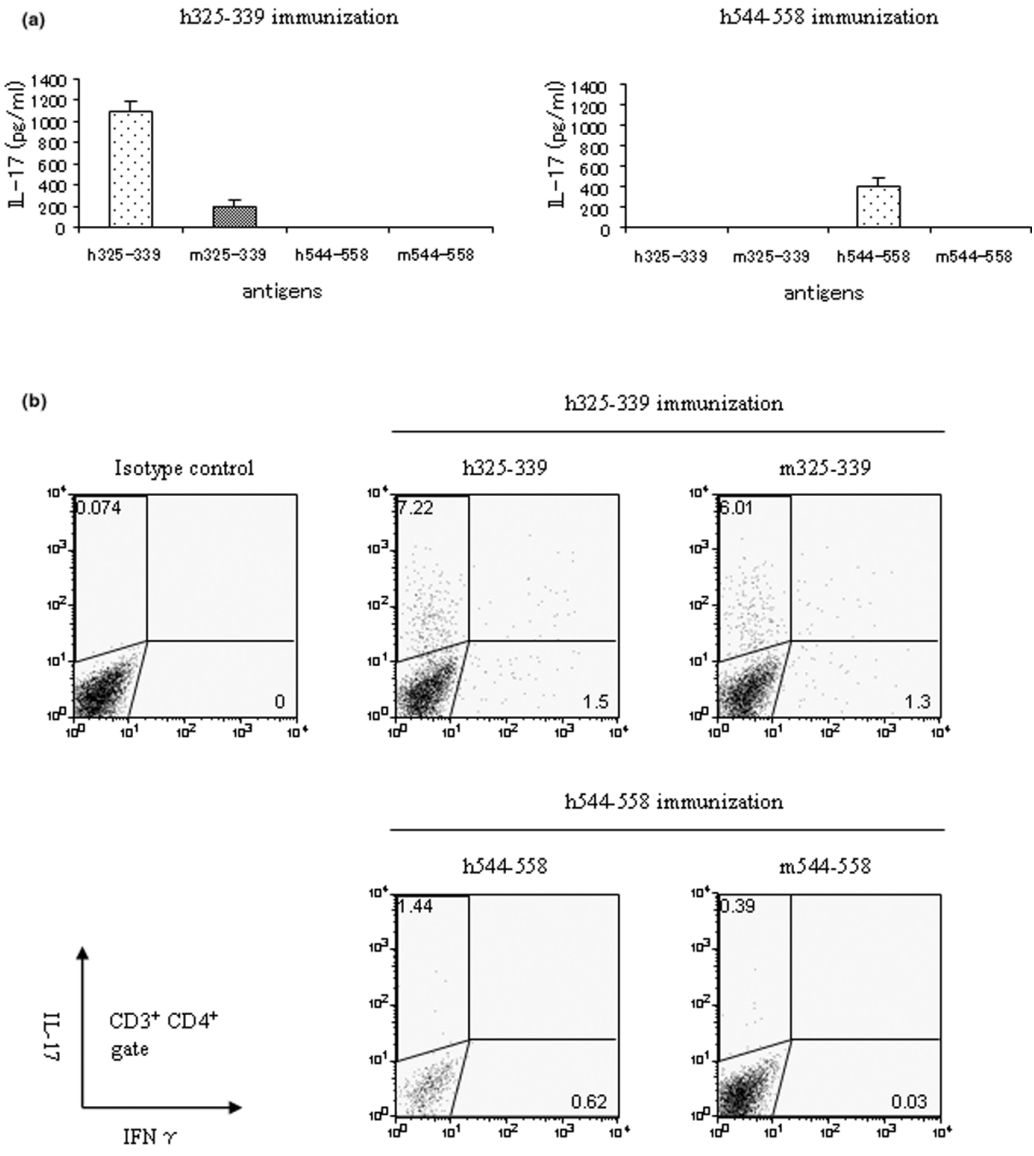
**Cross-reactivity with peptides derived from mouse glucose-6-phosphate isomerase (GPI)**. (a) Draining lymph node (DLN) cells taken from hGPI_325–339_-immunised mice on day 5 were cultured with 10 μM of hGPI_325–339_, mGPI_325–339_, hGPI_544–558 _or mGPI_544–558 _for 24 hours. The supernatants were assayed for interleukin (IL) 17 by ELISA. Data are averages ± standard deviation of three culture-wells. Representative data of three independent experiments. (b) DLN cells taken from hGPI_325–339_- or hGPI_544–558_-immunised mice on day 5 were cultured with 10 μM of hGPI_325–339 _and mGPI_325–339 _or hGPI_544–558 _and mGPI_544–558_, respectively. GoldiStop was added at the last four hours of each culture. Flow cytometry for IL-17 and interferon (IFN) γ was gated in CD3^+^, CD4^high ^cells. Representative flow cytometry data of three independent experiments with two mice per experiment.

First, we speculated that the difference in cross-reactivity to mouse GPI might affect the incidence of arthritis, because hGPI_325–339 _(IWYINCFGCETHAML) has 13/15 amino acids homology to mGPI_325–339 _(IWYINCYGCETHALL) while hGPI_544–558 _(GLINFIKQQREARVQ) has only 9/15 amino acids homology to mGPI_544–558 _(GLISFIKQQRDTKLE). The draining lymph node cells from mice immunised with hGPI_325–339 _or hGPI_544–558_were cultured in the presence of hGPI_325–339_, mGPI_325–339_, hGPI_544–558 _or mGPI_544–558 _for 24 hours. The hGPI_325–339_-primed cells had distinct cross-reactive immune reaction to mGPI_325–339 _by producing IL-17, whereas the hGPI_544–558 _primed cells did not cross-react to mGPI_544–558 _(Figure 5a). As compared with the draining lymph node cells of hGPI_325–339_-immunised mice, IL-17 production was not remarkable in that of hGPI_544–558_-immunised mice even when the corresponding peptide was used as an antigen for *in vitro *stimulation (Figure 5a). The production of IFN-γ was much lower than that of IL-17, and IL-4 production was not detectable independent of immunisation patterns and antigens for *in vitro *stimulation (data not shown).

It has been reported that Th17 cells are not the only cellular sources of IL-17, but CD8^+ ^T cells, natural killer T cells and γδT cells are also capable of producing IL-17 [17-22]. Therefore, we investigated the IL-17 producing cells using flow cytometry. The draining lymph node cells from mice immunised with hGPI_325–339 _or hGPI_544–558 _were stimulated with hGPI_325–339 _and mGPI_325–339_, or hGPI_544–558 _and mGPI_544–558_, respectively. Intracellular cytokine staining was performed without nonspecific stimulants, such as phorbol myristate acetate or ionomycin. We confirmed that immunisation of hGPI_325–339 _induced antigen-specific Th17 cells, which cross-reacted with mGPI_325–339_. However, immunisation of hGPI_544–558 _induced neither hGPI_544–558_-specific Th17 cells nor Th17 cells that can cross-react with mGPI_544–558 _remarkably (Figure 5b). These data indicate that induction of antigen-specific Th17 cells and cross-reactivity with mouse GPI might be the pathogenesis of peptide-induced arthritis.

### Immunisation of human GPI_325–339 _leads B cells to produce anti-mouse GPI antibodies

**Figure 6:**
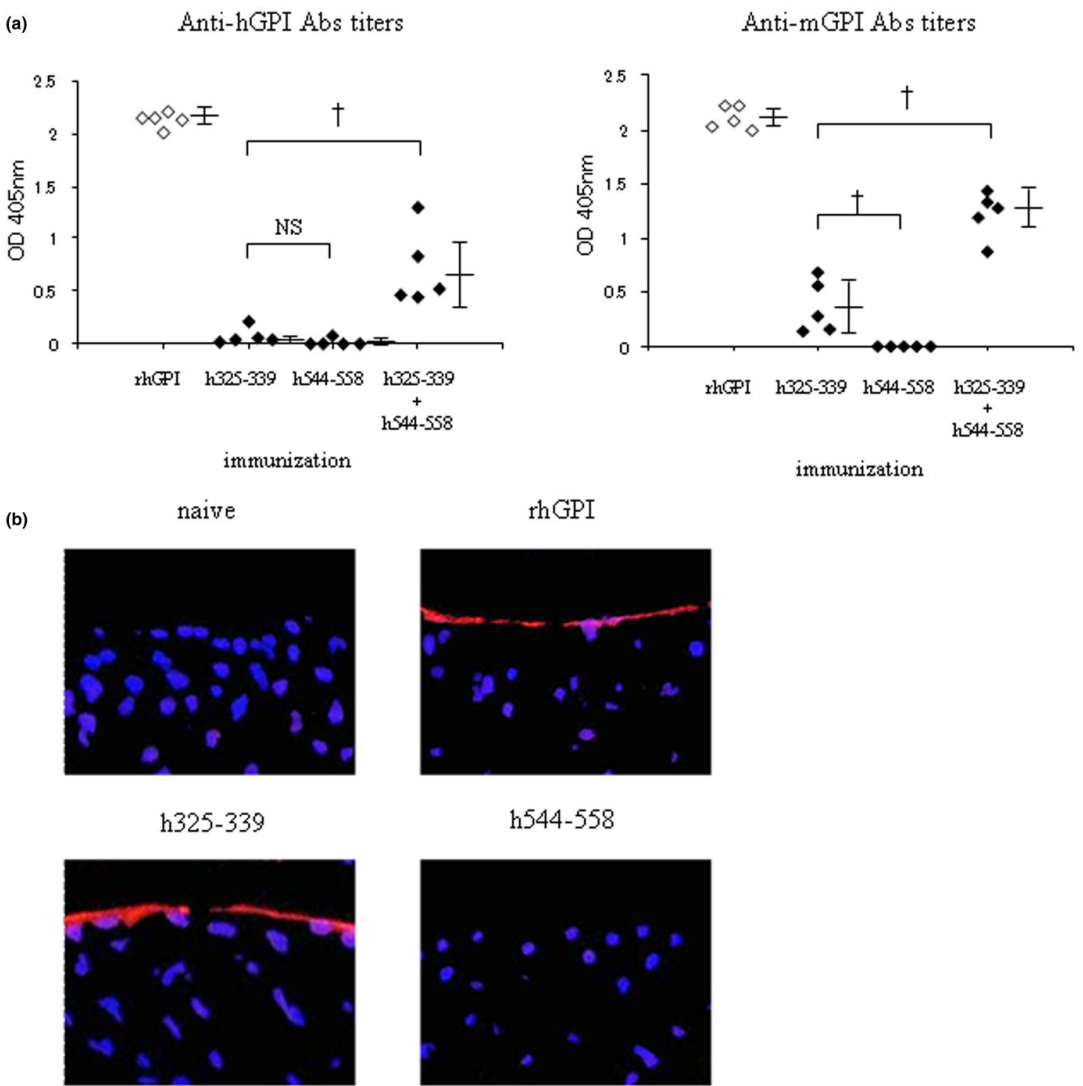
**Titres of anti-mouse glucose-6-phosphate isomerase (GPI) antibodies were elevated in mice with arthritis**. (a) Sera were taken on day 14 from mice immunised with recombinant human (rh) GPI, hGPI_325–339_, hGPI_544–558 _or hGPI_325–339 _plus hGPI_544–558_, and the titres of anti-human GPI antibodies and anti-mouse GPI antibodies were analysed by ELISA. Each symbol represents a single mouse. Data are mean optimal density ± standard deviation. †p < 0.01 (Mann-Whitney's U test). Representative data of two independent experiments. (b) Ankle joints were taken on day 14 from mice immunised with rhGPI, hGPI_325–339 _or hGPI_544–558_. Cryostat sections of ankle joints were stained with anti-mouse IgG (red), and nuclei were counterstained with 4',6-diamidino-2-phenyindole dilactate (blue). Representative data of three independent experiments.

To explore the importance of autoantibodies, we measured anti-human GPI antibodies and anti-mouse GPI antibodies in mice immunised with hGPI_325–339_, hGPI_544–558 _and hGPI_325–339 _plus hGPI_544–558_by ELISA. Mice immunised with rhGPI and the two peptides (hGPI_325–339 _plus hGPI_544–558_) produced high titres of anti-human GPI antibodies and anti-mouse GPI antibodies, and mice immunised with hGPI_325–339 _and hGPI_544–558 _hardly produced any anti-human GPI antibodies. However, mice immunised with hGPI_325–339 _produced significantly higher titres of anti-mouse GPI antibodies than mice immunised with hGPI_544–558 _(Figure 6a). It is noteworthy that immunisation with the two peptides (hGPI_325–339 _plus hGPI_544–558_) induced significantly higher titres of anti-mouse GPI antibodies than that with hGPI_325–339 _alone, whereas the severity and incidence of arthritis in mice immunised with two peptides (hGPI_325–339 _plus hGPI_544–558_) were comparable with those in mice immunised with hGPI_325–339 _alone (Figures 3a and 6a).

We further investigated the difference of the correlation between anti-mouse GPI antibodies and arthritis score among immunisation patterns. Each of the three different immunisation patterns (rhGPI, hGPI_325–339 _and hGPI_325–339 _plus hGPI_544–558_) showed no positive correlation between anti-mouse GPI antibodies and arthritis score (Table 5).

**Table 5:** Correlation between anti-mouse glucose-6-phosphate isomerase (GPI) antibodies titres and arthritis score

Immunisation	Rho value	P value
rhGPI	-0.825	0.0989
h325–339	-0.525	0.2937
h325–339 plus h544–558	0.500	0.3173

Sera were taken on day 14 from mice immunised with recombinant human (rh) GPI, hGPI_325–339 _or hGPI_325–339 _plus hGPI_544–558_. The correlation between the titres of anti-GPI antibodies and arthritis score on day 14 were statistically analysed with the Spearman's rank correlation coefficient. In the case of five samples, Rho values above 0.900 indicate significant positive correlation between anti-mouse GPI antibody titres and arthritis score, whereas Rho values below -0.900 indicate significant negative correlation (p < 0.05). Five mice per group. Representative data of two independent experiments.

Next, we investigated the existence of IgG on the cartilage surface by immunohistology, because GPI were proved to deposit on the cartilage surface of normal naïve mice [23]. The cryostat sections of ankle joints from naïve mice and mice immunised with hGPI_544–558 _did not show IgG deposit on the cartilage surface. However, those from mice immunised with rhGPI and hGPI_325–339 _showed IgG deposits (Figure 6b). These data indicate that anti-mouse GPI antibodies may play a role in the development of peptide-induced arthritis.

## Discussion

GPI, a ubiquitous glycolytic enzyme, is a new autoantigen candidate in autoimmune arthritis [5,6]. GPI-induced arthritis is induced by immunisation of genetically unaltered DBA/1 mice with rhGPI [1]. We report here the therapeutic efficacies of mAb to tumour necrosis factor-α and IL-6 and CTLA-4 Ig in this model [3]. Moreover, CD4^+ ^T cells, especially Th17 cells, seem to be more important than B cells, because administration of anti-CD4 mAb or anti-IL-17 mAb markedly ameliorate the progress of arthritis independent of anti-GPI antibodies titres [1,2]. Therefore, exploring the epitope of CD4^+ ^T cells and its arthritogenic effect is important for understanding the pathological mechanisms.

In this study, we investigated the binding motif of I-A^q ^from T cell epitopes considered to bind to I-A^q^, synthesised peptides of epitope candidates and identified hGPI_325–339 _as a major epitope. Interestingly, the MHC binding residues of hGPI_325–339_(IWYINCFGCETHAML) at P1, P4 and P7 were the same as those for bovine CII_256–270 _(GEPGIAGFKGEQGPK), the dominant epitope of collagen-induced arthritis [4]. These findings indicate that the binding motif (P1 I, P4 F, P7 E) might have high binding affinity with I-A^q^, and the peptides with this motif-MHC complexes might be effectively recognised by TCRs and could be arthritogenic in some condition. Although immunisation with a fragment of cyanogen bromide of bovine CII, CB11 (CII_124–402_), which contains the dominant epitope, can induce arthritis, the severity and incidence are much lower than arthritis induced by bovine CII protein [4]. Other fragments (CB8, CB9, CB10 and CB12) do not induce arthritis, as is explained by the production of anti-bovine CII antibodies. Immunisation with CB11 fragment produces five times more antibodies to bovine CII than any other fragment [4]. The observation that administration of anti-CD4 mAb after the onset of arthritis did not ameliorate the arthritis [24,25] and a combination of mAb to CII can passively transfer arthritis to naïve mice [26] also emphasises the importance of autoantibodies to the induction of collagen-induced arthritis.

Our study demonstrated that immunisation with hGPI_325–339 _induced antigen-specific Th17 cells, which can cross-react with mGPI_325–339 _and lead B cells to produce anti-mouse GPI antibodies. However, immunisation with hGPI_544–558 _could not even induce hGPI_544–558_-specific Th17 cells. The difference of ability of Th17 induction between two peptides may come from MHC-binding affinity and TCR-binding affinity. A peptide that is likely to bind to MHC class II with high affinity and interacts strongly with the T cell receptor tends to stimulate Th1-cell response, whereas a peptide with low binding affinity to MHC class II and T cell receptor tends to elicit Th2-cell response [27,28]. Although the relationship between Th17 differentiation and the strength of TCR signalling and MHC-binding affinity has not been clarified, it is possible that the difference in amino acid sequences between hGPI_325–339 _and hGPI_544–558 _might affect the I-Aq binding affinity and the TCR signalling, and consequently lead to the difference in extent of antigen-specific Th17 cells. In this study, we did not detect any IL-4 production, which is an adjuvant effect of *Mycobacterim tuberculosis *and pertussis toxin.

In K/BxN mice expressing I-A^g7 ^as MHC class II molecules, mGPI_282–294_-specific CD4^+ ^T cells lead B cells to produce anti-mouse GPI antibodies [16]. The anti-mouse GPI antibodies from K/BxN mice have such high affinity that IgG transfer of K/BxN mice can provoke arthritis in normal mice [6]. In comparison, the anti-mouse GPI antibodies from GPI-induced arthritis alone are not sufficient for the development of arthritis because IgG transfer from mice immunised with rhGPI can not provoke arthritis. However, IgG signalling through FcγR seems necessary for the induction of GPI-induced arthritis because FcγR-deficient mice are resistant to arthritis [1]. Moreover, the data that transfer of rhGPI-primed or hGPI_325–339_-primed Th17 cells to naïve DBA/1 mice can not induce arthritis emphasises the necessity of anti-mouse GPI antibodies (unpublished observation). Considering the data that there are no positive correlation between anti-mouse GPI antibodies and arthritis score [[29] and unpublished observation], and arthritis-resistant mice like C57BL/6 produce as high titres of anti-mouse GPI antibodies as DBA/1 when immunised with rhGPI (1 and unpublished observation), anti-mouse GPI antibodies may play a subordinate role in the development of GPI-induced arthritis and peptide-induced arthritis in DBA/1 mice.

In the process of epitope screening, the response to hGPI_539–558 _peptide was comparable with that to hGPI_327–346 _peptide; however, the response to hGPI_542–556 _and hGPI_544–558_, which were synthesised with 90% purity, was lower than that to hGPI_539–558 _peptide. Furthermore, the response to hGPI_539–558_, which was re-synthesised with 90% purity, was much lower than to hGPI_325–339 _or to hGPI_539–558 _peptide for screening (data not shown). These results could be explained by differences in the purity of the synthetic peptides. The synthetic peptides used for screening (peptides numbers 1 to 25, Table 2) were unpurified, and the purity of each peptide would have been quite different, although the exact purity was unchecked by the product maker. Therefore, it is possible that the purity of number 25 peptide might have been much higher than that of number 18 peptide, or alternatively, number 25 peptide may have contained other peptides through peptide synthesis.

From a probability point of view, it is possible that other epitopes exist in some regions of human GPI-amino acid sequence from which we did not synthesise the peptides, because I-A^q ^may have another binding motif and our synthesised peptides covered only the 399/558 (71.5%) amino acid residues of human GPI protein, not the whole length. However, two experimental pieces of data support that hGPI_325–339 _may be the dominant epitope. One is that immunisation with hGPI_325–339 _provoked arthritis similar to that induced by rhGPI protein. The other is that intraperitoneal injection of hGPI_325–339 _after the onset of arthritis significantly ameliorated the progress of arthritis (data not shown). Because systemic administration of a dominant epitope leads to anergy of pathogenic T cells or results in activation-induced cell death [30,31], this inhibitory effect of hGPI_325–339 _on GPI-induced arthritis supports the notion that hGPI_325–339 _may be the dominant epitope.

Cross-reactivity is considered the one of mechanisms of autoimmune diseases. We previously identified patients with RA who have GPI-reactive CD4^+ ^T cells and found that some of them express human leucocyte antigen-DR4 as MHC class II [32]. Because the I-A^q ^binding motif resembles DR4 [9], further studies are needed to define epitopes of CD4^+ ^T cells in such patients and search proteins that have homology to the epitopes.

## Conclusions

This study is the first report of experimental arthritis induced by immunisation with a single short peptide in genetically unaltered mice. The fact that an immunological reaction to a single short peptide of ubiquitously expressed protein causes polyarthritis provides new insight to the understanding of autoimmune arthritis.

## Abbreviations

APC: antigen-presenting cell; CIA: collagen-induced arthritis; CII: type II collagen; CTLA-4 Ig: cytotoxic T-lymphocyte antigen 4 immunoglobulin; DAPI: 4',6-diamidino-2-phenyindole, dilactate; ELISA: enzyme-linked immunosorbent assay; FCS: fetal calf serum; GPI: glucose-6-phosphate isomerase; IFN: interferon; IL: interleukin; mAb: monoclonal antibody; MHC: major histocompatibility complex; PBS: phosphate-buffered saline; RA: rheumatoid arthritis; rh: recombinant human; SD: standard deviation; SEM: standard error of the mean; TCR: T cell receptor; Th: T helper.

## Competing interests

The authors declare that they have no competing interests.

## Authors' contributions

KI wrote the manuscript and conceived of the study. YT and AI assisted experiments and statistical analysis. IM and TS participated in its full design and coordination, and DG, SI and AK participated in discussions.

## References

[B1] Schubert D, Maier B, Morawietz L, Krenn V, Kamradt T (2004). Immunization with glucose-6-phosphate isomerase induces T cell-dependent peripheral polyarthritis in generally unaltered mice. J Immunol.

[B2] Iwanami K, Matsumoto I, Tanaka-Watanabe Y, Mihira M, Ohsugi Y, Mamura M, Goto D, Ito S, Tsutsumi A, Kishimoto T, Sumida T (2008). Crucial role of IL-6/IL-17 axis in the induction of arthritis by glucose-6-phosphate isomerase. Arthritis Rheum.

[B3] Matsumoto I, Zhang H, Yasukochi T, Iwanami K, Tanaka Y, Inoue A, Goto D, Ito S, Tsutsumi A, Sumida T (2008). Therapeutic effects of antibodies to tumor necrosis factor-α, interleukin-6 and cytotoxic T-lymphocyte antigen 4 immunoglobulin in mice with glucose-6-phosphate isomerase induced arthritis. Arthritis Res Ther.

[B4] Brand DD, Myers LK, Terato K, Whittington KB, Stuart JM, Kang AH, Rosloniec EF (1994). Characterization of the T cell determinants in the induction of autoimmune arthritis by bovine α1(II)-CB11 in H-2^q ^mice. J Immunol.

[B5] Matsumoto I, Lee DM, Goldbach-Mansky R, Sumida T, Hitchon CA, Schur PH, Anderson RJ, Coblyn JS, Weinblatt ME, Brenner M, Duclos B, Pasquali JL, El-Gabalawy H, Mathis D, Benoist C (2003). Low prevalence of antibodies to glucose-6-phosphate isomerase in patients with rheumatoid arthritis and a spectrum of other chronic autoimmune disorders. Arthritis Rheum.

[B6] Matsumoto I, Staub A, Benoist C, Mathis D (1999). Arthritis provoked by linked T and B recognition of a glycolytic enzyme. Science.

[B7] MHCPred. http://www.jenner.ac.uk/MHCPred/.

[B8] Kawamoto T (2003). Use of a new adhesive film for the preparation of multi-purpose fresh-frozen sections from hard tissues, whole-animals, insects and plants. Arch Histol Cytol.

[B9] Bayrak Ş, Holmdahl R, Travers P, Lauster R, Hesse M, Dölling R, Mitchison NA (1997). T cell response of I-A^q ^mice to self type II collagen: meshing of the binding motif of the I-A^q ^molecule with repetitive sequences results in autoreactivity to multiple epitopes. Int Immunol.

[B10] Chen JS, Lorenz RG, Goldberg J, Allen PM (1991). Identification and characterization of a T cell-inducing epitope of bovine ribonuclease that can be restricted by multiple class II molecules. J Immunol.

[B11] Fritz RB, Skeen MJ, Chou CH, Garcia M, Egorov IK (1985). Major histocompatibility complex-linked control of the murine immune response to myelin basic protein. J Immunol.

[B12] Sakai K, Sinha AA, Mitchell DJ, Zamvil SS, Rothbard JB, McDevitt HO, Steinmann L (1988). Involvement of distinct murine T-cell receptors in the autoimmune encephalitogenic response to nested epitopes of myelin basic protein. Proc Natl Acad Sci USA.

[B13] Myers LK, Cooper SW, Terato K, Seyer JM, Stuart JM, Kang AH (1995). Identification and characterization of a tolerogenic T cell determinant within residues 181–209 of chick type II collagen. Clin Immunol Immunopathol.

[B14] Michaëlsson E, Andersson M, Engström A, Holmdahl R (1992). Identification of an immunodominant type-II collagen peptide recognized by T cells in H-2^q ^mice: self tolerance at the level of determinant selection. Eur J Immunol.

[B15] Myers LK, Seyer JM, Stuart JM, Terato K, David CS, Kang AH (1993). T cell epitopes of type II collagen that regulate murine collagen-induced arthritis. J Immunol.

[B16] Basu D, Horvarh S, Matsumoto I, Fremont DH, Allen PM (2000). Molecular basis for recognition of an arthritic peptide and a foreign epitope on distinct MHC molecules by a single TCR. J Immunol.

[B17] Bettelli E, Carrier Y, Gao W, Korn T, Strom TB, Oukka M, Weiner HL, Kuchroo VK (2006). Reciprocal development pathways for the generation of pathogenic effector T_H_17 and regulatory T cells. Nature.

[B18] Mangan PR, Harrington LE, O'Quinn DB, Helms WS, Bullard DC, Elson CO, Hatton RD, Wahl SM, Schoeb TR, Weaver CT (2006). Transforming growth factor-beta induces development of the T (H) 17 lineage. Nature.

[B19] He D, Wu L, Kim HK, Li H, Elmets CA, Xu H (2006). CD8+ IL-17 producing T cells are important effector functions for the elicitation of contact hypersensitivities responses. J Immunol.

[B20] Michel ML, Keller AC, Paget C, Fujio M, Trottein F, Savage PB, Wong CH, Schneider E, Dy M, Leite-de-Moraes MC (2007). Identification of an IL-17-producing NK1.1neg iNKT cell population involved in airway neutrophilia. J Exp Med.

[B21] Yoshiga Y, Goto D, Segawa S, Ohnishi Y, Matsumoto I, Ito S, Tsutsumi A, Taniguchi M, Sumida T (2008). Invariant NKT cells produce IL-17 through IL-23-dependent and -independent pathways with potential modulation of Th17 response in collagen-induced arthritis. Int J Mol Med.

[B22] Lockhart E, Green AM, Flynn JL (2006). IL-17 production is dominated by gammadelta T cells rather than CD4 T cells during Mycobacterium tuberculosis infection. J Immunol.

[B23] Matsumoto I, Maccioni M, Lee DM, Maurice M, Simmons B, Brenner M, Mathis D, Benoist C (2002). How antibodies to a ubiquitous cytoplasmic enzyme may provoke joint-specific autoimmune disease. Nat Immunol.

[B24] Ranges GE, Sriram S, Cooper SM (1985). Prevention of type II collagen-induced arthritis by in vivo treatment with anti-L3T4. J Exp Med.

[B25] Williams RO, Whyte A (1996). Anti-CD4 monoclonal antibodies suppress murine collagen-induced arthritis only at the time of primary immunization. Cell Immunol.

[B26] Terato K, Harper DS, Griffiths MM, Hasty DL, Ye XJ, Cremer MA, Seyer JM (1995). Collagen-induced arthritis in mice: synergistic effect of E. coli lipopolysaccharide bypasses epitope specificity in the induction of arthritis with monoclonal antibodies to type II collagen. Autoimmunity.

[B27] Constant S, Pfeiffer C, Woodard A, Pasqualini T, Bottomly K (1995). Extent of T cell receptor ligation can determine the functional differentiation of naïve CD4+ T cells. J Exp Med.

[B28] Leitenberg D, Boutin Y, Constant S, Bottomly K (1998). CD4 regulation of TCR signaling and T cell differentiation following stimulation with peptides of different affinities for the TCR. J Immunol.

[B29] Bockermann R, Schubert D, Kamradt T, Holmdahl R (2005). Induction of a B-cell-dependent chronic arthritis with glucose-6-phosphate isomerase. Arthritis Res Ther.

[B30] Critchfield JM, Racke MK, Zúòiga-Pflücker JC, Cannellla B, Raine CS, Goverman J, Lenardo MJ (1994). T cell deletion in high antigen dose therapy of autoimmune encephalomyelitis. Science.

[B31] Gaur A, Wiers B, Liu A, Rothbard J, Fathman CG (1992). Amelioration of autoimmune encephalomyelitis by myelin basic protein synthetic peptide-induced anergy. Science.

[B32] Kori Y, Matsumoto I, Zhang H, Yasukochi T, Hayashi T, Iwanami K, Goto D, Ito S, Tsutsumi A, Sumida T (2006). Characterisation of Th1/Th2 type, glucose-6-phosphate isomerase reactive T cells in the generation of rheumatoid arthritis. Ann Rheum Dis.

